# Amoxicillin or tetracycline in bismuth-containing quadruple therapy for *Helicobacter pylori* eradication: a systematic review and meta-analysis

**DOI:** 10.3389/fmicb.2025.1667516

**Published:** 2025-09-19

**Authors:** Kunping Ju, Qingzhou Kong, Shanling Zhang, Liangrun Zhu, Yueyue Li

**Affiliations:** ^1^Department of Gastroenterology, Qilu Hospital of Shandong University, Jinan, Shandong, China; ^2^Department of Gastroenterology, Qingdao third People's Hospital Affiliated to Qingdao University, Qingdao, China

**Keywords:** amoxicillin, bismuth-containing quadruple therapy, efficacy, *Helicobacter pylori*, tetracycline

## Abstract

**Background:**

Amoxicillin and tetracycline have been widely used in *Helicobacter pylori* (*H. pylori*) eradication therapy, and the priority of their efficacy and safety in Bismuth-containing Quadruple Therapy (BQT) remain controversial.

**Materials and methods:**

A comprehensive systematic review was conducted by searching databases from their inception until June 2025. Studies that compared BQT arms containing amoxicillin with those containing tetracycline were included. Pooled Relative Risks (RR) and 95% Confidence Intervals (CI) of the efficacy and safety outcomes were reported.

**Results:**

Seven randomized controlled trials and two observational studies were included in the meta-analysis. The pooled eradication rates of amoxicillin-containing BQT versus tetracycline-containing BQT in the first-line treatment were not statistically different in the intention-to-treat (86.5% vs. 81.4%, RR: 1.07, 95% CI: 0.99−1.17, *P* = 0.10) and per-protocol (93.3% vs. 90.7%, RR: 1.03, 95% CI: 0.97−1.10, *P* = 0.34) analyses. The eradication rates for rescue therapy yielded similar results in the ITT (81.1% vs. 89.7%, RR: 0.90, 95% CI: 0.79−1.03, *P* = 0.13) and PP (85.1% vs. 93.4%, RR: 0.91, 95% CI: 0.80−1.03, *P* = 0.14) analyses. The risk of total adverse events was lower in the amoxicillin-containing BQT than in the tetracycline group (26.8% vs. 37.1%, *P* < 0.00001). No difference in total compliance (94.8% vs. 92.8%, *P* = 0.20).

**Conclusion:**

The efficacy of both amoxicillin- and tetracycline-containing BQT demonstrated comparable eradication rates and compliance, while the amoxicillin group exhibited fewer adverse events.

## Introduction

*Helicobacter pylori* infection occurs in nearly half of the global population and is closely related to the incidence of gastric cancer (Thrift et al., 2023; Li et al., 2023). The combination of appropriate antibiotic regimen can cure *H. pylori* infection, and the eradication is effective in reducing the morbidity and mortality of gastric cancer (Yan et al., 2022; Li et al., 2019). However, a global challenge remains in the effective and safe treatment of *H. pylori*.

Nowadays, High-Dose Dual Therapy (HDDT) and Bismuth-containing Quadruple Therapy (BQT) are widely recommended as first-line treatment regimens, with tetracycline and metronidazole being the classic antibiotics combination for the latter (Malfertheiner et al., 2022; Chey et al., 2024). In addition, amoxicillin/metronidazole, amoxicillin/tetracycline, amoxicillin/clarithromycin, amoxicillin/levofloxacin, etc. are also commonly used antibiotic combinations in quadruple therapies (Zhou et al., 2022). However, tetracycline is not clinically available in many countries (Tian et al., 2023), and the risk of common adverse effects at high doses and complicated administration (three or four times daily) reduce patient compliance (Nyssen et al., 2021). Against this background, modified bismuth-containing quadruple regimens emerged (Zhou et al., 2022). The prevalence of *H. pylori* resistance reported for amoxicillin, the backbone of eradication therapy, remains low globally (Savoldi et al., 2018). Also, amoxicillin has advantages of clinical accessibility and affordable price (Tian et al., 2023). Recently, several randomized controlled studies have compared amoxicillin or tetracycline in bismuth-containing quadruple regimens for *H. pylori* eradication therapy. However, to date, no meta-analysis has been conducted to provide a pooled analysis of the relevant evidence (Tian et al., 2023; Chen et al., 2016).

Hence, we performed a systematic review and meta-analysis to compare the efficacy and safety of amoxicillin- or tetracycline-containing bismuth quadruple therapy.

## Materials and methods

### Database and literature search strategy

The study was registered in PROSPERO (number: CRD42024527090) and followed a PRISMA statement (Page et al., 2021). PubMed, Embase, Web of Science, and the Cochrane Library were searched until June 2025 for studies comparing amoxicillin or tetracycline in bismuth-containing quadruple therapy. The following search terms were used: “*Helicobacter pylori, H. pylori, Hp*,” “amoxicillin, amoxycillin” and “tetracycline”. Language restrictions were not imposed during the search process. The details of the search strategy for each database are as presented in Supplementary Table 1.

### Study selection

Two reviewers (JKP and KQZ) independently screened the studies. The abstracts or full manuscripts of all studies identified by the literature search were reviewed and selected based on the following selection criteria. When disagreements arose, further verification was conducted until a consensus was reached.

### Inclusion criteria

The inclusion criteria for the meta-analysis were based on the PICOS principle. (P) Participants: patients with *H. pylori* infection diagnosed by one of the following tests: ^13^C-/^14^C-urea breath test, rapid urease test, histological examination, or *H. pylori* culture. Both first-line and rescue (failed one or more courses of eradication therapies previously) treatment patients were included. (I) Intervention: amoxicillin-containing bismuth quadruple regimen group receiving Proton Pump Inhibitors (PPIs) or potassium-competitive acid blockers (P-CABs), bismuth, amoxicillin and another antibiotic. (C) Comparator: tetracycline-containing bismuth quadruple regimen group receiving PPIs or P-CABs, bismuth, tetracycline and another antibiotic identical to that used in the amoxicillin-containing regimen. (O) Outcomes: (i) primary outcome: *H. pylori* eradication rate; (ii) secondary outcome: adverse events and compliance. (S) Study design: randomized controlled trials (RCTs) and observational studies.

### Exclusion criteria

The exclusion criteria are as follows: (1) studies using sequential therapy contaminated with a dual-therapy regimen, (2) studies without antibiotics in the study protocol, and (3) studies without exact dosage or frequency of medication.

### Data extraction

Two reviewers (JKP and KQZ) independently extracted data using a predesigned data extraction form. The following variables were extracted from each trial: author, year of publication, country of study, study design, participant characteristics, treatment line, and specific details of the eradication regimen (name, dose, frequency, and duration). Others include diagnostic criteria for confirming *H. pylori* infection before and after eradication, antibiotic resistance, data related to eradication rates, adverse events, and compliance. The third author addressed any discrepancies.

### Risk of bias assessment

Two reviewers (JKP and KQZ) independently assessed the risk of bias of the RCTs using the Cochrane Collaboration's Risk of Bias Assessment Tool (Sterne et al., 2019). The Newcastle-Ottawa scale (NOS) was used to assess observational studies (cohort studies, case-control studies) (Stang, 2010). A consensus was reached between the two reviewers to resolve any differences.

### Statistical analysis

We calculated the pooled Relative Risks (RR) and 95% Confidence Intervals (CI) for each study via meta-analysis using a random-effects model. Intention-to-treat (ITT) analysis and per-protocol (PP) analysis of *H. pylori* eradication rates was performed for data analysis. The index of heterogeneity (*I*^2^) was calculated by Chi-square test to measure heterogeneity, with *I*^2^ ≥50% indicating a high degree of variability between studies. This analysis performed sensitivity and subgroup analyses to investigate the sources of heterogeneity when *I*^2^ results were ≥ 50%. Publication bias was omitted as only 9 studies were included (Sterne et al., 2011). All analyses were performed using Review Manager software (version 5.4, Cochrane Collaboration, Nordic Cochrane Centre, Copenhagen, Denmark) and STATA/SE 17.0 (STATA Inc., Texas, USA). Differences between groups at *P* < 0.05 were considered statistically significant.

## Results

### Study selection and characteristics

**Figure 1:**
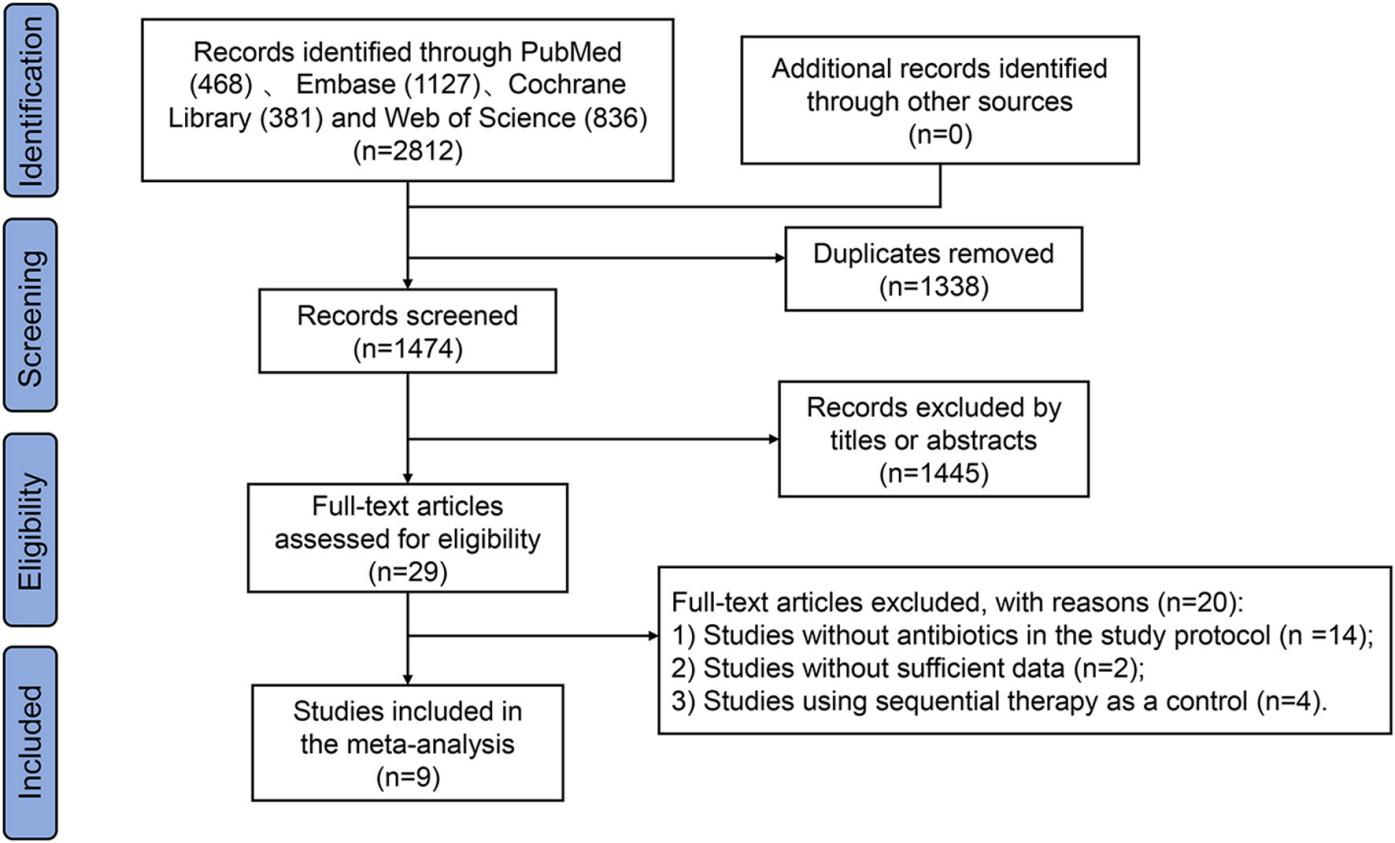
PRISMA diagram of the literature search.

This meta-analysis identified a total of 2812 studies as shown in the flow diagram (Figure 1). Of the 2812 studies identified from PubMed (*n* = 468), Embase (*n* = 1127), Cochrane Library (*n* = 381), and Web of Science (*n* = 836), 1338 were removed for duplication. Another one thousand four hundred and forty five studies were excluded by titles or abstracts, and 20 were removed after full-text review (RCTs without antibiotics in the study protocol = 14, studies without sufficient data = 2, and studies using sequential therapy as a control = 4). Finally, the study enrolled nine studies (Uygun et al., 2008; Liang et al., 2013; Chen et al., 2016; Salmanroghani et al., 2018; Bang et al., 2020; Yozgat et al., 2021; Hsu et al., 2021; Tian et al., 2023; Xie et al., 2025), including 2287 patients infected with *H. pylori*. Out of these, one thousand hundred and seventy nine (47.2%) patients were assigned to the amoxicillin-containing regimen group and one thousand two hundred and eight (52.8%) to the tetracycline-containing regimen group.

The study characteristics are summarized in Table 1. The enrolled studies included 7 RCTs (Uygun et al., 2008; Liang et al., 2013; Chen et al., 2016; Salmanroghani et al., 2018; Bang et al., 2020; Hsu et al., 2021; Tian et al., 2023) and 2 observational studies (Yozgat et al., 2021; Xie et al., 2025), all geographically restricted to Asian populations. The therapeutic regimens were employed as first-line *H. pylori* eradication therapy in 4 studies (Salmanroghani et al., 2018; Bang et al., 2020; Yozgat et al., 2021; Tian et al., 2023) and as rescue therapy in the remaining 5 studies (Uygun et al., 2008; Liang et al., 2013; Chen et al., 2016; Hsu et al., 2021; Xie et al., 2025). All studies analyzed three outcomes: eradication rate, adverse events, and compliance, except for one study (Uygun et al., 2008) that lacked data on adverse events.

**Table 1:** Characteristics of the studies enrolled in the meta-analysis.

**Study**	**Country**	**Study design**	**Patient number**	**Indication**	**Treatment line**	**Diagnostic criterion**	**Assessed after treatment**	**Regimen**	**ITT**	**PP**	**Lost to follow-up**
Uygun, 2007	Turkey	RCT	200	Non-ulcer dyspepsia	Rescue therapy	Both histology and RUT	Histology and RUT	L 30 mg bid, B 300 mg qid, M 500 mg bid, A 1 g bid (14 days)	68/100(68.0%)	68/91(74.7%)	NA
								L 30 mg bid, B 300 mg qid, M 500 mg bid, T 500 mg qid (14 days)	78/100(78.0%)	78/95(82.1%)	NA
Liang, 2013	China	RCT	212	Functional dyspepsia and scarred peptic ulcers	Rescue therapy	RUT and culture, or ^13^C-UBT	^13^C-UBT	L 30 mg bid, B 220 mg bid, F 100 mg tid, A 1 g tid (14 days)	99/104(95.2%)	99/100(99.0%)	0
								L 30 mg bid, B 220 mg bid, F 100 mg tid, T 500 mg qid (14 days)	99/108(91.7%)	99/103(96.1%)	1/108(0.9%)
Chen, 2016	China	RCT	312	*NA*	Rescue therapy	^13^C-UBT and at least one of the three tests (RUT, histology, and culture)	^13^C-UBT	L 30 mg bid, B 220 mg bid, M 400 mg qid, A 1 g tid (14 days)	138/156(88.5%)	133/142(93.7%)	7/156(4.5%)
								L 30 mg bid, B 220 mg bid, M 400 mg qid, T 500 mg qid (14 days)	136/156(87.2%)	122/128(95.3%)	6/156(3.8%)
Salmanroghani, 2018	Iran	RCT	228	Duodenal ulcer	First-line	RUT and endoscopy	^13^C-UBT	O 20 mg bid, B 240mg tid, M 500mg tid, A 1g tid (14 days)	105/113(92.9%)	105/110(95.5%)	2/113 (1.8%)
								O 20 mg bid, B 240mg qid, M 500mg tid, T 500mg qid (14 days)	88/115(76.5%)	88/105(83.8%)	4/115(3.5%)
Bang, 2020	Korea	RCT	233	NA	First-line	RUT, ^13^C-UBT, or histology	^13^C-UBT	R 20 mg bid, B 300 mg qid, M 500 mg tid, A 1 g bid (14 days)	102/117(87.2%)	101/105(96.2%)	11/117(9.4%)
								R 20 mg bid, B 300 mg qid, M 500 mg tid, T 500 mg qid (14 days)	96/116(82.8%)	94/97(96.9%)	16/116 (13.8%)
Yozgat, 2020	Turkey	Retrospective study	244	NA	First-line	Histology	*NA*	P 40 mg bid, B 262 mg qid, M 500 mg tid, A 1 g bid (14 days)	90/102 (88.2%)	90/94 (95.7%)	NA
								P 40 mg bid, B 262 mg qid, M 500 mg tid, T 500 mg qid (14 days)	116/142 (81.7%)	116/128 (90.6%)	NA
Hsu, 2021	China	RCT	112	*NA*	Rescue therapy	(1) ^13^C-UBT, (2) both RUT and histology, or (3) culture	^13^C-UBT	E 40 mg bid, B 300 mg qid, L 500 mg qd, A 500 mg qid (10 days)	39/56 (69.6%)	37/53 (69.8%)	1/56 (1.8%)
								E 40 mg bid, B 300 mg qid, L 500 mg qd, T 500 mg qid (10 days)	50/56 (89.3%)	49/55 (89.1%)	0
Tian, 2022	China	RCT	404	Dyspepsia	first-line	(1) ^13^C-UBT or (2) both RUT and histology	^13^C-UBT	E 20 mg bid, B 110 mg qid, M 400 mg qid, A 500 mg qid (14 days)	165/202(81.7%)	161/181(89.0%)	2/202 (1.0%)
								E 20 mg bid, B 110 mg qid, M 400 mg qid, T 500 mg qid (14 days)	168/202(83.2%)	163/178(91.6%)	3/202 (1.5%)
Xie, 2025	China	Retrospective study	342	NA	Rescue therapy	^13^C or ^14^C-UBT or histology	^13^C or ^14^C-UBT or histology	E 20 mg bid (or P 40 mg bid or R 10 mg/20 mg bid or V 20 mg bid), B 220 mg bid, F 100 mg bid, A 1000 mg bid (10 or 14 days)	98/129(76.0%)^*^	98/125(77.8%)	73/202 (36.1%)
								E 20 mg bid (or P 40 mg bid or R 10 mg/20 mg bid or V 20 mg bid), B 220 mg bid, F 100 mg bid, T 500 mg tid (10 or 14 days)	205/213(96.2%)^*^	201/207(97.1%)	169/382 (44.2%)

A, amoxicillin; B, bismuth; bid, twice a day; E, esomeprazole; F, furazolidone; ITT, intention-to-treat analysis; L, lansoprazole; L, levofloxacin; M, metronidazole; NA, not available; O, omeprazole; P, pantoprazole; PP, per-protocol analysis; qd, one time a day; qid, four times a day; R, rabeprazole; RCT, randomized controlled trial; RUT, rapid urease test; T, tetracycline; tid, three times a day; UBT, urea breath test; V, vonoprazan. ^*^mITT, modified intention-to-treat analysis.

### Amoxicillin versus tetracycline in bismuth quadruple regimen

#### Eradication rate

**Figure 2:**
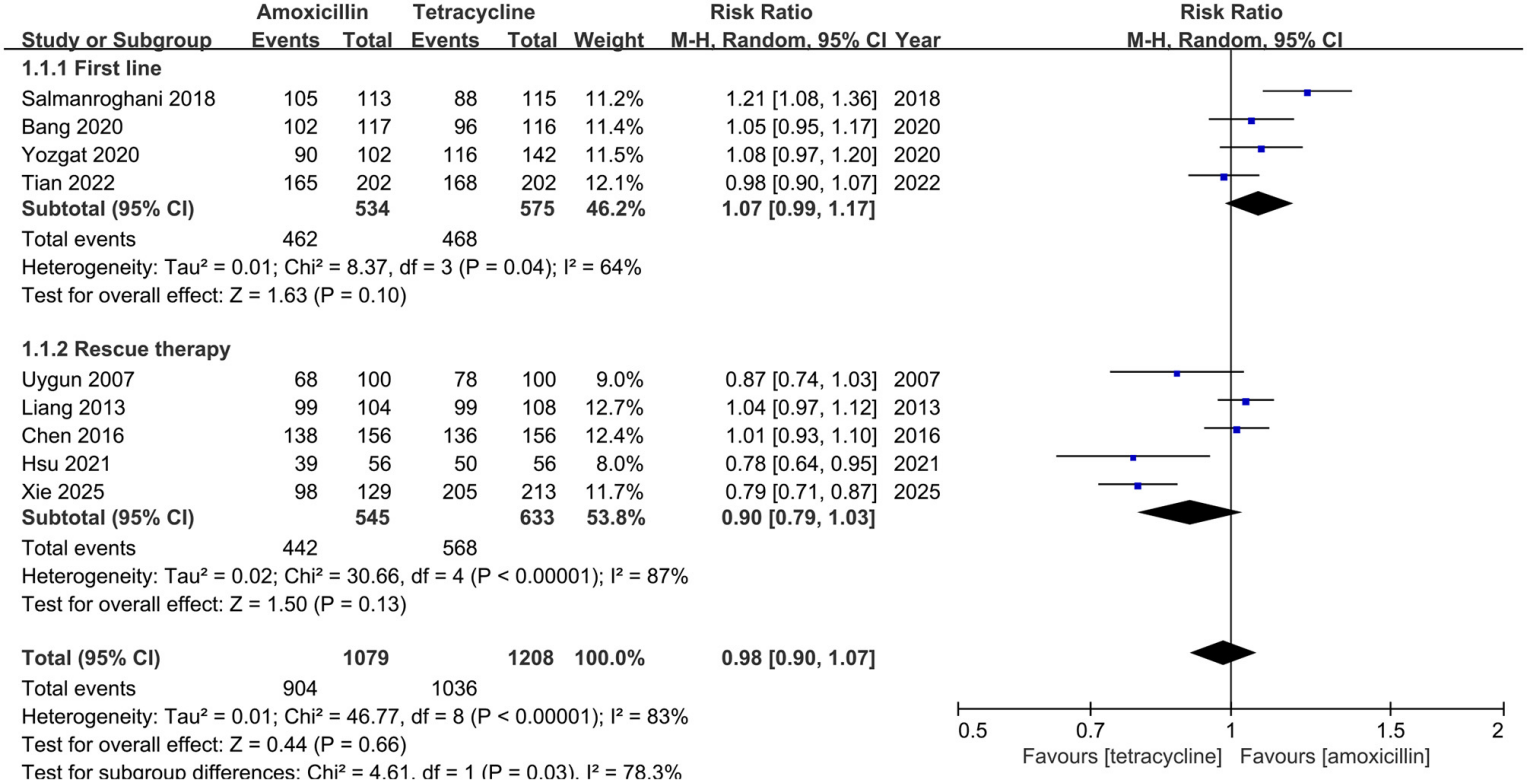
Forest plot for eradication rate comparison between amoxicillin-containing therapy and tetracycline-containing therapy according to ITT analysis.

**Figure 3:**
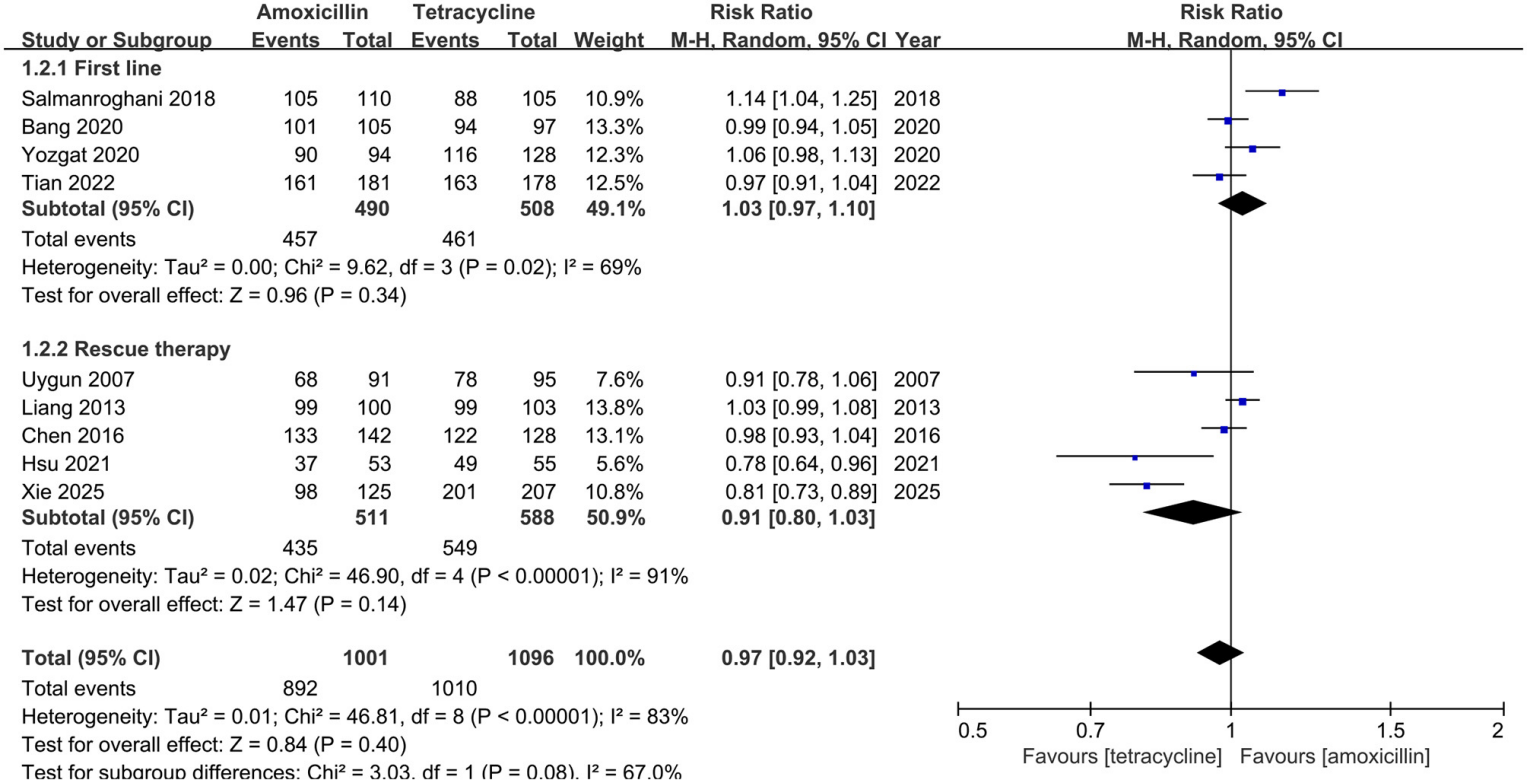
Forest plot for eradication rate comparison between amoxicillin-containing therapy and tetracycline-containing therapy according to PP analysis.

In ITT analysis, amoxicillin-containing and tetracycline-containing bismuth quadruple regimens achieved similar efficacy (83.8% vs. 85.8%, RR: 0.98, 95% CI: 0.90–1.07, *P* = 0.66) with statistically significant heterogeneity (*I*^2^ = 83%, *P* < 0.00001; Figure 2). In PP analysis, amoxicillin-containing and tetracycline-containing bismuth quadruple regimens also achieved similar efficacy (89.1% vs. 92.2%, RR: 0.97, 95% CI: 0.92–1.03, *P* = 0.40) with statistically significant heterogeneity (*I*^2^ = 83%, *P* < 0.00001; Figure 3).

#### Subgroup analysis

Based on the treatment line (first-line or rescue therapy) in ITT analysis, the eradication rate for treatment-naïve patients in the amoxicillin-containing group was similar to the tetracycline-containing group (86.5% vs. 81.4%, RR: 1.07, 95% CI: 0.99–1.17, *P* = 0.10; *I*^2^ = 64%, *P* = 0.04; Figure 2). For the retreatment patients, the eradication rates in the two groups were also similar (81.1% vs 89.7%, RR: 0.90, 95% CI: 0.79–1.03, *P* = 0.13; *I*^2^ = 87%, *P* < 0.00001; Figure 2). In the PP analysis, there was no significant difference in the eradication rates between the two groups of naïve (93.3% vs. 90.7%, RR: 1.03, 95% CI: 0.97–1.10, *P* = 0.34; *I*^2^ = 69%, *P* = 0.02; Figure 3) or retreatment patients (85.1% vs 93.4%, RR: 0.91, 95% CI: 0.80–1.03, *P* = 0.14; *I*^2^ = 91%, *P* < 0.00001; Figure 3).

Stratified by study design, the eradication rates of amoxicillin-containing versus tetracycline-containing groups showed no significant differences in either RCTs (ITT: 84.4% vs. 83.8%, RR: 1.01, 95% CI: 0.93–1.09, *P* = 0.88; *I*^2^ = 72%, *P* = 0.002; PP: 90.0% vs. 91.1%, RR: 0.99, 95% CI: 0.95–1.05, *P* = 0.82; *I*^2^ = 69%, *P* = 0.004; Supplementary Figure 1) or observational studies (ITT: 81.4% vs. 90.4%, RR: 0.92, 95% CI: 0.68–1.26, *P* = 0.61; *I*^2^ = 94%, *P* < 0.0001; PP: 85.8% vs 94.6%, RR: 0.93, 95% CI: 0.69–1.24, *P* = 0.60; *I*^2^ = 96%, *P* < 0.00001; Supplementary Figure 2).

In addition, five studies (Bang et al., 2020; Chen et al., 2016; Tian et al., 2023; Hsu et al., 2021; Xie et al., 2025) reported *H. pylori* eradication rates in the presence of antimicrobial resistance (Supplementary Table 2). No significant difference in the eradication rates between the two groups was observed in the subgroup analysis stratified by amoxicillin (68.2% vs 89.3%, RR: 0.84, 95% CI: 0.54–1.32, *P* = 0.45; *I*^2^ = 40%, *P* = 0.17; Supplementary Figure 3), tetracycline resistance (90.9% vs. 80.0%, RR: 1.01, 95% CI: 0.76–1.35, *P* = 0.92; *I*^2^ = 0%, *P* = 0.87; Supplementary Figure 4), metronidazole (93.2% vs. 93.6%, RR: 1.00, 95% CI: 0.95–1.05, *P* = 0.97; *I*^2^ = 0%, *P* = 0.66; Supplementary Figure 5), levofloxacin (93.1% vs 95.9%, RR: 0.99, 95% CI: 0.90–1.09, *P* = 0.84; *I*^2^ = 55%, *P* = 0.11; Supplementary Figure 6), and clarithromycin (97.3% vs. 96.8%, RR: 1.01, 95% CI: 0.96–1.05, *P* = 0.82; *I*^2^ = 0%, *P* = 0.97; Supplementary Figure 7).

#### Sensitivity analysis

In the sensitivity analysis performed by observing the outlier based on the forest plot and excluding the studies conducted by Salmanroghani et al. (Salmanroghani et al., 2018), Hsu *et al*. (Hsu et al., 2021) and Xie *et al*. (Xie et al., 2025), the *I*^2^ was dramatically reduced to 18% and 36% in the ITT and PP analyses, respectively, indicating that these three studies may be a source of statistical heterogeneity.

#### Adverse events

**Figure 4:**
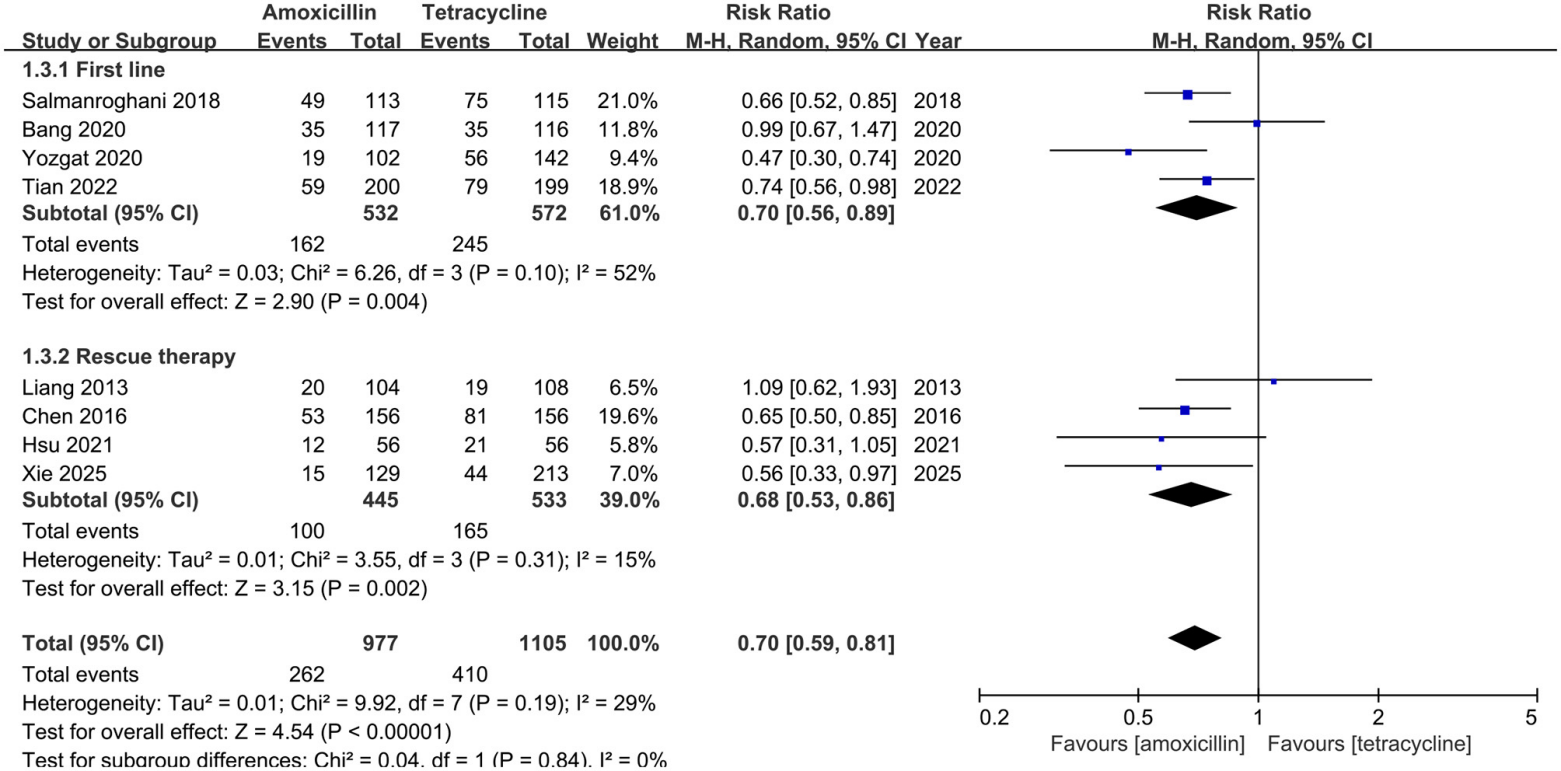
Forest plot for adverse events comparison between amoxicillin-containing therapy and tetracycline-containing therapy.

Eight studies recorded data on adverse events. As shown in Figure 4, the adverse events of amoxicillin-containing group are significantly lower than the tetracycline-containing group (26.8% vs. 37.1%, RR: 0.70, 95% CI: 0.59–0.81, *P* < 0.00001). The details and severity of the adverse events are presented in Supplementary Table 3. In further analysis, the amoxicillin-containing group demonstrated significantly lower rates of nausea/vomiting (12.4% vs. 19.2%, RR: 0.59, 95% CI: 0.44–0.80, *P* = 0.0006; Supplementary Figure 8) and dizziness (4.1% vs. 7.7%, RR: 0.56, 95% CI: 0.38–0.84, *P* = 0.005; Supplementary Figure 9) compared to the tetracycline-containing group. However, both groups showed comparable rates of abdominal pain (3.8% vs. 6.6%, RR: 0.65, 95% CI: 0.38–1.10, *P* = 0.11; Supplementary Figure 10), diarrhea (3.8% vs 4.6%, RR: 0.82, 95% CI: 0.48–1.42, *P* = 0.48; Supplementary Figure 11), abnormal taste (7.8% vs 9.0%, RR: 0.83, 95% CI: 0.63–1.09, *P* = 0.17; Supplementary Figure 12), headache (3.5% vs. 4.4%, RR: 0.85, 95% CI: 0.51–1.42, *P* = 0.54; Supplementary Figure 13), fatigue (4.3% vs 5.6%, RR: 0.75, 95% CI: 0.50–1.13, *P* = 0.17; Supplementary Figure 14), and skin rash (2.6% vs. 2.4%, RR: 1.07, 95% CI: 0.61–1.86, *P* = 0.82; Supplementary Figure 15).

#### Compliance

**Figure 5:**
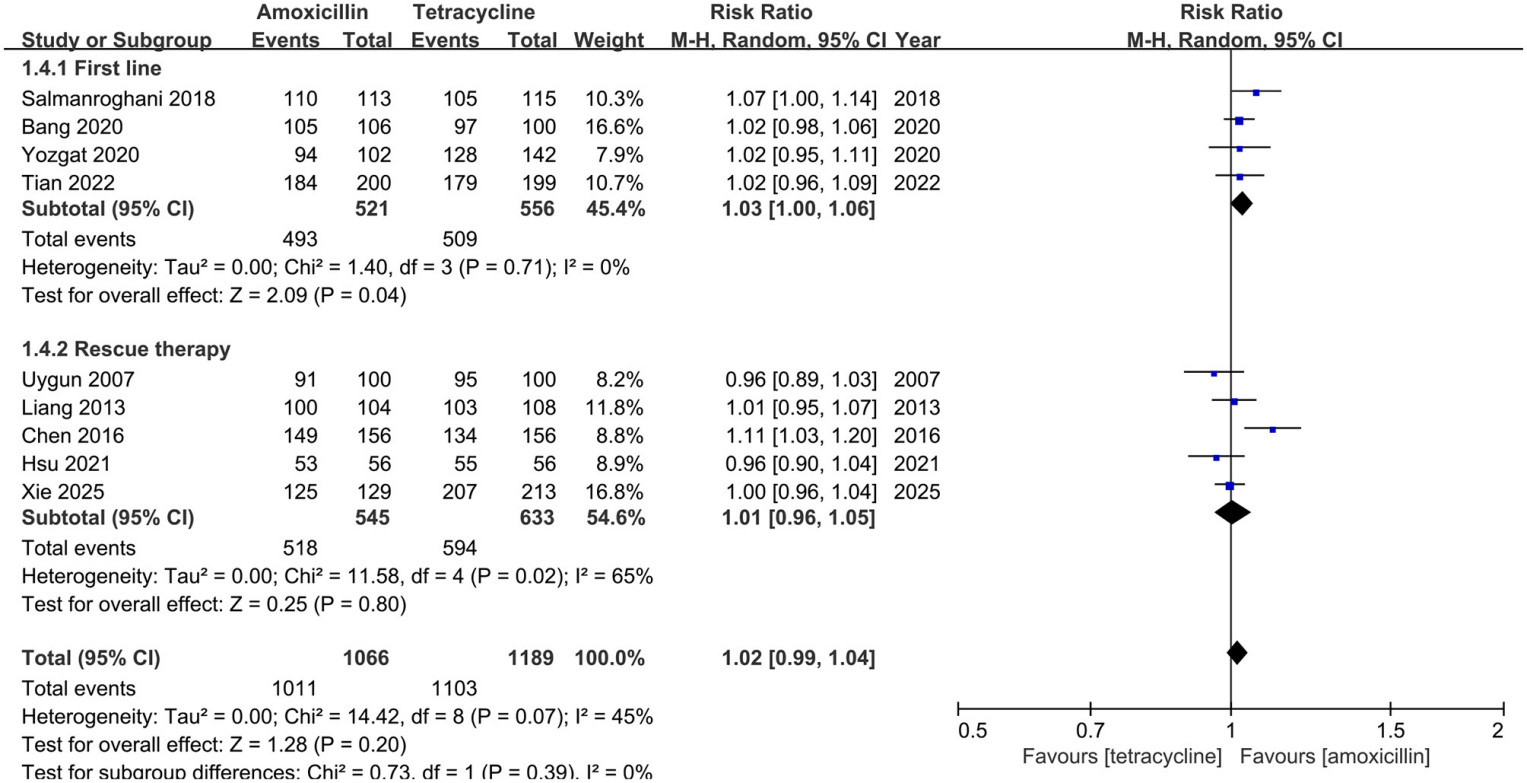
Forest plot for compliance comparison between amoxicillin-containing therapy and tetracycline-containing therapy.

All the enrolled studies provided compliance information. As shown in Figure 5, the compliance of amoxicillin-containing group is similar to that of tetracycline-containing group (94.8% vs. 92.8%, RR: 1.02, 95% CI: 0.99–1.04, *P* = 0.20).

### Risk of bias assessment

For seven RCTs, none of the domains or the overall judgment had a high risk-of-bias. Two observational studies scored 7 or higher and were judged to be of high quality. Other details of the assessment are provided in Supplementary Table 4,5.

## Discussion

This is the first meta-analysis which systematically analyzed the efficacy of tetracycline- and amoxicillin-containing quadruple therapies. Eradication rates, adverse events, and compliance were compared between the two groups.

In the meta-analysis of nine studies, no significant difference in the eradication rate was observed between the two groups by ITT and PP analysis. The results may be dependent on the current low resistance to tetracycline and amoxicillin (Xie et al., 2018). Moreover, eradication rates of over 90 % were achieved despite metronidazole resistance (Supplementary Figure 5). No significant difference was observed in eradication rates between the two groups, indicating that high doses of metronidazole could partially overcome metronidazole resistance (Fallone et al., 2016). The application of bismuth may also improve the eradication rates in cases of metronidazole resistance (Han et al., 2022). The antimicrobial mechanisms of bismuth against gastrointestinal pathogens, including *H. pylori*, involved inhibition of protein synthesis, cell wall formation, membrane function, and ATP production (Lambert and Midolo, 1997). Another mechanistic study likewise supported a direct inhibitory effect of bismuth on *H. pylori*, including the disruption of motility and metabolic pathways associated with bacterial growth (Yao et al., 2021).

Notably, tetracycline-containing bismuth quadruple regimen had an eradication rate of only 81.4 % in first-line therapy, according to the ITT analysis, which was not in line with what has been reported in the literature (Liou et al., 2016; Tursi et al., 2017). The low eradication rates may be related to the low compliance in the included first-line therapy studies (*P* = 0.04), the latter of which may be attributed to the high rate of adverse events (*P* = 0.004). Further studies are warranted to validate the findings.

There was high observed heterogeneity of results during the analysis of the eradication rate. Based on the random-effects model, further subgroup and sensitivity analyses were refined to explore the reasons for the heterogeneity. No clear cause of heterogeneity was revealed by subgroup analysis stratified by treatment time and antimicrobial resistance. Furthermore, in the sensitivity analysis, a significant drop was observed in *I*^2^ when the studies conducted by Salmanroghani et al. (2018); Hsu et al. (2021); and Xie et al. (2025) were excluded. This observed reduction may be related to the population characteristics, such as duodenal ulcers patients in the first study, suggesting a possible source of heterogeneity in the subject population. Furthermore, tetracycline resistance rates in the latter two studies were both 0%. Particularly, in the study by Xie et al. (0% for tetracycline resistance vs. 29.6% for amoxicillin resistance), the apparent difference may also contribute to the heterogeneity in eradication rates between the two regimens.

Classic bismuth quadruple therapy has a high extra-therapeutic effect, with up to 37% adverse effects (Nyssen et al., 2021). In our study, the amoxicillin-containing regimen treatment group had lower incidence of adverse events than the tetracycline-containing regimen treatment group (*P* < 0.00001), which may depend on the safety profile of amoxicillin (Nyssen et al., 2021). Even when amoxicillin was administered at a dose of 3g per day, no significant adverse events occurred (Chen et al., 2016; Salmanroghani et al., 2018). However, there was no difference in compliance between the two groups (*P* = 0.20), which may be related to the equally good eradication rate of the tetracycline-containing regimen. In addition, larger sample size and more high-quality clinical studies are needed to assess compliance in both groups.

Optimization of *H. pylori* eradication therapies should be based on the principle of benefit to primary outcome, and regimens with a cure rate of less than 90% should be discarded (Graham and Liou, 2022). Since bismuth quadruple therapy containing amoxicillin or tetracycline both achieved acceptable eradication rates for first-line *H. pylori* eradication in PP analyses (93.3% vs. 90.7%), secondary outcome metrics can be taken into consideration for a more comfortable treatment experience. When penicillin allergy is ruled out, amoxicillin-containing regimens may be preferred with the advantages of fewer adverse events and no specific concerns for use in the pediatric population.

This study had some limitations. First, most studies were conducted in Asian countries. Further validation is required to determine the influence of racial differences on the efficacy of these two regimens. Second, heterogeneity exists when data from different studies are combined and analyzed. Several factors may have contributed to heterogeneity, including the included subjects, drug dosage, and evaluation of results. Third, biases, such as a lack of allocation concealment and blinding of participants and personnel, exist in these studies. The lack of blinding might have influenced the reporting of side effects (Gao et al., 2020). Fourth, the number of included studies is relatively small, with the risk that a lack of significance could result from this. Larger populations and more high-quality studies are needed for further validation. Finally, only five studies reported the results of antimicrobial sensitivity detection, possibly with bias caused by the small sample sizes.

## Conclusion

In conclusion, our analysis demonstrated that the amoxicillin-containing bismuth quadruple regimen was as effective as the tetracycline-containing bismuth quadruple regimen in eradicating *H. pylori*, with fewer adverse events and similar compliance, whether for patients undergoing initial or rescue therapies. Subgroup analysis stratified by antimicrobial resistance support the conclusion that the two groups had similar efficacy.
