# Supplementary material for: Amoxicillin or tetracycline in bismuth-containing quadruple therapy for Helicobacter pylori eradication: a systematic review and meta-analysis
Source: Front Microbiol. 2025 Sep 19;16:1667516. doi: 10.3389/fmicb.2025.1667516 (PMC12492358; doi:10.3389/fmicb.2025.1667516)
Supplement: Supplementary file 1 [file Table_1.docx]

| **Supporting online information** | | |
| --- | --- | --- |
| **Content** | | **Page** |
| **Table S1.** | Search strategies | 3-4 |
| **Table S2.** | Antibiotic resistance rates of included studies | 5 |
| **Table S3.** | Treatment-related adverse events during eradication therapy | 5-6 |
| **Table S4.** | Risk of bias assessment for randomized controlled trials included on the basis of Cochrane Collaboration's Risk of Bias Assessment Tool 2 (RoB 2) | 7 |
| **Table S5.** | Quality assessment for cohort studies included on the basis of Newcastle-Ottawa quality assessment scale | 7 |
| **Figure S1**. | Forest plot of subgroup analysis stratified by study design in ITT analysis | 8 |
| **Figure S2**. | Forest plot of subgroup analysis stratified by study design in PP analysis | 8 |
| **Figure S3**. | Forest plot of subgroup analysis stratified by amoxicillin resistance | 9 |
| **Figure S4**. | Forest plot of subgroup analysis stratified by tetracycline resistance | 9 |
| **Figure S5**. | Forest plot of subgroup analysis stratified by metronidazole resistance | 9 |
| **Figure S6**. | Forest plot of subgroup analysis stratified by levofloxacin resistance | 9 |
| **Figure S7**. | Forest plot of subgroup analysis stratified by clarithromycin resistance | 9 |
| **Figure S8**. | Forest plot for the incidence of nausea/vomiting between amoxicillin-containing therapy and tetracycline-containing therapy | 10 |
| **Figure S9**. | Forest plot for the incidence of dizziness between amoxicillin-containing therapy and tetracycline-containing therapy | 10 |
| **Figure S10**. | Forest plot for the incidence of abdominal pain between amoxicillin-containing therapy and tetracycline-containing therapy | 11 |
| **Figure S11**. | Forest plot for the incidence of diarrhea between amoxicillin-containing therapy and tetracycline-containing therapy | 11 |
| **Figure S12**. | Forest plot for the incidence of abnormal taste between amoxicillin-containing therapy and tetracycline-containing therapy | 12 |
| **Figure S13**. | Forest plot for the incidence of headache between amoxicillin-containing therapy and tetracycline-containing therapy | 12 |
| **Figure S14**. | Forest plot for the incidence of fatigue between amoxicillin-containing therapy and tetracycline-containing therapy | 13 |
| **Figure S15**. | Forest plot for the incidence of skin rash between amoxicillin-containing therapy and tetracycline-containing therapy | 13 |

**Table S1. Search strategies (from inception to June 2025)**

| **Databases** | **Search strategies** | **Results** |
| --- | --- | --- |
| **PubMed** | (((("Amoxicillin"[Mesh]) OR (((Amoxicillin[Title/Abstract]) OR (Amoxycillin[Title/Abstract])) OR (Amoxicilline[Title/Abstract]))) AND (("Tetracycline"[Mesh]) OR (((Tetracycline[Title/Abstract]) OR (Acheomycin[Title/Abstract])) OR (Achromycin[Title/Abstract])))) AND (("Helicobacter pylori"[Mesh]) OR ((((((Helicobacter pylori[Title/Abstract]) OR (Helicobacter nemestrinae[Title/Abstract])) OR (Campylobacter pylori[Title/Abstract])) OR (H. pylori[Title/Abstract])) OR (H.pylori[Title/Abstract])) OR (Hp[Title/Abstract])))) AND ((quadruple[Title/Abstract]) OR (bismuth[Title/Abstract])) | **468** |
| **EMBASE** | #1 'amoxicillin'/exp OR 'amoxicillin':ab,ti OR 'amoxycillin':ab,ti OR 'amoxicilline':ab,ti  #2 'tetracycline'/exp OR 'tetracycline':ab,ti OR 'acheomycin':ab,ti OR 'achromycin':ab,ti  #3 'helicobacter'/exp OR 'helicobacter':ab,ti OR 'campylobacter pylori':ab,ti OR 'h. pylori':ab,ti OR 'h.pylori':ab,ti OR 'hp':ab,ti  #4 'quadruple':ab,ti OR 'bismuth':ab,ti  **#5** **#1 AND #2 AND #3 AND #4** | 91442  121447  125669  24376  **1127** |
| **Cochrane library** | #1 MeSH descriptor: [Amoxicillin] explode all trees  #2 (Amoxicillin OR Amoxycillin OR Amoxicilline):ti,ab,kw  #3 #1 OR #2  #4 MeSH descriptor: [Tetracyclines] explode all trees  #5 (Tetracyclines OR Tetracycline OR Acheomycin OR Achromycin):ti,ab,kw  #6 #4 OR #5  #7 MeSH descriptor: [Helicobacter pylori] explode all trees  #8 (Helicobacter pylori OR Helicobacter nemestrinae OR Campylobacter pylori OR H. pylori OR H.pylori OR Hp):ti,ab,kw  #9 #7 OR #8  #10 (quadruple OR bismuth):ti,ab,kw  **#11 #3 AND #6 AND #9 AND #10** | 3592  7344  7344  3129  2519  4403  2630  9668  9668  3149  **381** |
| **Web of**  **Science** | #1 ((TS=(Amoxicillin)) OR TS=(Amoxycillin)) OR TS=(Amoxicilline)  #2 ((TS=(Tetracycline)) OR TS=(Acheomycin)) OR TS=(Achromycin)  #3 (((((TS=(Helicobacter pylori)) OR TS=(Helicobacter nemestrinae)) OR TS=(Campylobacter pylori)) OR TS=(H. pylori)) OR TS=(H.pylori)) OR TS=(Hp)  #4 (TS=(quadruple)) OR TS=(bismuth)  **#5 #1 AND #2 AND #3 AND #4** | 40217  107053  181924  205321  **836** |

**Table S2. Antibiotic resistance rates of included studies**

| Study | Regimen | Amoxicillin | Tetracycline | Metronidazole | Levofloxacin | Clarithromycin |
| --- | --- | --- | --- | --- | --- | --- |
| Bang 2020 | A-M 14 days | 6.5% (6/93) | 9.7% (9/93) | 32.3% (30/93) | 39.8% (37/93) | 23.9% (28/93) |
|  | T-M 14 days | 10.3% (9/87) | 9.2% (8/87) | 48.3% (42/87) | 44.8% (39/87) | 22.4% (26/87) |
| Chen 2016 | A-M 14 days | 8.2% (9/110) | 0.9% (1/110) | 87.3% (96/110) | 88.2% (97/110) | 90.0% (99/110) |
|  | T-M 14 days | 8.4% (8/95) | 1.1% (1/95) | 88.4% (84/95) | 82.1% (78/95) | 90.5% (86/95) |
| Hsu 2021 | A-L 10 days | 0 | 0 | - | 23.8% (5/21) | - |
|  | T-L 10 days | 0 | 0 | - | 50.0% (8/16) | - |
| Tian 2022 | A-M 14 days | 4.9% (3/61) | 4.9% (3/61) | 63.9% (39/61) | 37.7% (23/61) | 32.8% (20/61) |
|  | T-M 10 days | 4.4% (3/68) | 5.4% (4/68) | 64.7% (44/68) | 32.4% (22/68) | 32.4% (22/68) |
| Xie 2025 | A-F 10 or 14 days | 40.0% (4/10) | 0 | - | - | - |
|  | T-F 10 or 14 days | 27.3% (12/44) | 0 | - | - | - |

**Table S3. Treatment-related adverse events during eradication therapy**

| **Study** | **Patient number** | **Regimen** | **Bloating** | **Nausea/**  **vomiting** | **Abdominal pain** | **Diarrhea** | **Constipa-tion** | **Skin rash** | **Headache** | **Fatigue** | **Fever** | **Abnormal taste** | **Dizziness** | **mild** | **moderate** | **severe** | **Dropouts due to AE** |
| --- | --- | --- | --- | --- | --- | --- | --- | --- | --- | --- | --- | --- | --- | --- | --- | --- | --- |
| Liang,  2013 | 212 | A-F  14 days | - | 1.9% (2/104) | 1.0% (1/104) | 1.0% (1/104) | - | 5.8% (6/104) | 1.0% (1/104) | 4.8% (5/104) | 1.0% (1/104) | 1.0% (1/104) | 1.0% (1/104) | - | - | - | 3.8% (4/104) |
|  |  | T-F  14 days | - | 2.8% (3/108) | 0 | 0.9% (1/108) | - | 1.9% (2/108) | 1.9% (2/108) | 6.5% (7/108) | 1.9% (2/108) | 0 | 1.9% (2/108) | - | - | - | 3.7% (4/108) |
| Chen,  2016 | 312 | A-M  14 days | 5.1% (8/156) | 21.8% (34/156) | 0.6% (1/156) | 0.6% (1/156) | - | 1.9% (3/156) | 1.3% (2/156) | 1.3% (2/156) | 0.6% (1/156) | 1.3% (2/156) | 6.4% (10/156) | 19.2% (30/156) | 9.6% (15/156) | 5.1% (8/156) | 5.1% (8/156) |
|  |  | T-M  14 days | 3.8% (6/156) | 38.5% (60/156) | 2.6% (4/156) | 4.5% (7/156) | - | 1.3% (2/156) | 0.6% (1/156) | 3.8% (6/156) | 1.3% (2/156) | 2.6% (4/156) | 16.0% (25/156) | 25.0% (39/156) | 16.0% (25/156) | 10.9% (17/156) | 10.3% (16/156) |
| Salmanroghani,  2018 | 228 | A-M  14 days | - | 8.0% (9/113) | - | 5.3% (6/113) | - | 3.5% (4/113) | - | 1.8% (2/113) | 1.8% (2/113) | 16.8% (19/113) | 2.7% (3/113) | - | - | - | - |
|  |  | T-M  14 days | - | 18.3% (21/115) | - | 7.0% (8/115) | - | 3.5% (4/115) | - | 6.1% (7/115) | 2.6% (3/115) | 19.1% (22/115) | 3.5% (4/115) | - | - | - | - |
| Bang,  2020 | 233 | A-M  14 days | - | 17.1% (20/117) | 6.0% (7/117) | 6.8% (8/117) | - | 0 | 4.3% (5/117) | 3.4% (4/117) | - | 1.7% (2/117) | 2.6% (3/117) | 25.6% (30/117) | 3.4% (4/117) | 0.9% (1/117) | - |
|  |  | T-M  14 days | - | 16.4% (19/116) | 4.3% (5/116) | 2.6% (3/116) | - | 0.9% (1/116) | 3.4% (4/116) | 2.6% (3/116) | - | 3.4% (4/116) | 8.6% (10/116) | 19.8% (23/116) | 6.9% (8/116) | 3.4% (4/116) | - |
| Yozgat,  2020 | 244 | A-M  14 days | - | 8.8% (9/102) | 5.8% (6/102) | - | - | 3.9% (4/102) | 7.8% (8/102) | - | - | 11.7% (15/102) | 8.8% (9/102) | - | - | - | - |
|  |  | T-M  14 days | - | 18.3% (26/142) | 16.9% (24/142) | - | - | 5.6% (8/142) | 9.8% (14/142) | - | - | 14.7% (21/142) | 9.8% (14/142) | - | - | - | - |
| Hsu,  2021 | 112 | A-L  10 days | - | 5.4% (3/56) | 3.6% (2/56) | 10.7% (6/56) | 0 | 1.8% (1/56) | 3.6% (2/56) | 3.6% (2/56) | - | 5.4% (3/56) | 1.8% (1/56) | - | - | - | 5.4% (3/56) |
|  |  | T-L  10 days | - | 30.4% (17/56) | 3.6% (2/56) | 8.9% (5/56) | 1.8% (1/56) | 0 | 1.8% (1/56) | 5.4% (3/56) | - | 5.4% (3/56) | 7.1% (4/56) | - | - | - | 1.8% (1/56) |
| Tian,  2022 | 404 | A-M  14 days | - | 20.0% (40/200) | 5.5% (11/200) | 5.0% (10/200) | 1.5% (3/200) | 3.5% (7/200) | 4.0% (8/200) | 10.0% (20/200) | - | 16.0% (32/200) | 4.0% (8/200) | 13.5% (27/200) | 11.0% (22/200) | 5.0% (10/200) | 3.5% (7/200) |
|  |  | T-M  10 days | - | 25.1% (50/199) | 8.0% (16/199) | 7.5% (15/199) | 1.5% (3/199) | 3.5% (7/199) | 6.0% (12/199) | 12.6% (25/199) | - | 21.6% (43/199) | 5.0% (10/199) | 17.1% (34/199) | 14.6% (29/199) | 8.0% (16/199) | 4.5% (9/199) |
| Xie,  2025 | 342 | A-F 10 or 14 days |  | 3.1% (4/129) |  | 0.8% (1/129) | 0 | 0 |  | 2.3% (3/129) |  | 1.5% (2/129) |  | 10.9% (14/129) | 0.8% (1/129)* | | 0.8% (1/129) |
|  |  | T-F 10 or 14 days |  | 7.5% (16/213) |  | 2.3% (5/213) | 0.5% (1/213) | 0.9% (2/213) |  | 1.4% (3/213) |  | 1.4% (3/213) |  | 17.8% (38/213) | 2.8% (6/213)* | | 2.3% (5/213) |

**Abbreviations:** A, amoxicillin; AE, adverse events; F, furazolidone; L, levofloxacin; M, metronidazole; T, tetracycline.

*Moderate/Severe adverse events.

**Table S4. Risk of bias assessment for randomized controlled trials included on the basis of Cochrane Collaboration's Risk of Bias Assessment Tool 2 (RoB 2)**

| **Study** | **Bias arising from the randomization process** | **Bias due to deviations from intended interventions** | **Bias due to missing outcome data** | **Bias in measurement of the outcome** | **Bias in selection of the reported result** | **Overall judgment** |
| --- | --- | --- | --- | --- | --- | --- |
| Bang 2020 | + | ? | + | + | + | ? |
| Chen 2016 | + | + | + | + | + | + |
| Hsu 2021 | + | + | + | + | + | + |
| Liang 2013 | + | + | + | + | + | + |
| Salmanroghani 2018 | + | ? | + | + | + | ? |
| Tian 2022 | + | + | + | + | + | + |
| Uygun 2007 | + | ? | + | + | + | ? |

**Note: +** = low risk of bias, ? =some concerns, - = high risk of bias.

Overall judgment criteria: Low risk of bias: The study is judged to be at low risk of bias for all domains for this result; Some concerns: The study is judged to raise some concerns in at least one domain for this result, but not to be at high risk of bias for any domain; High risk of bias: The study is judged to be at high risk of bias in at least one domain for this result, or the study is judged to have some concerns for multiple domains in a way that substantially lowers confidence in the result.

**Table S5.** **Quality assessment for cohort studies included on the basis of Newcastle-Ottawa quality assessment scale**

|  | **Selection**  (score) | | | | **Comparability**  (score) | **Outcome**  (score) | | | **Total Score** |
| --- | --- | --- | --- | --- | --- | --- | --- | --- | --- |
|  | Representativeness of the exposed cohort | Selection of the non exposed cohort | Ascertainment of exposure | Demonstration that outcome of interest was not present at start of study | Comparability of cohorts on the basis of the design or analysis | Assessment of outcome | Was follow-up long enough for outcomes to occur | Adequacy of follow up of cohorts |  |
| Yozgat, 2020 | 1 | 0 | 1 | 1 | 2 | 0 | 1 | 1 | 7 |
| Xie, 2025 | 1 | 0 | 1 | 1 | 2 | 1 | 1 | 1 | 8 |

**Figure S1. Forest plot of subgroup analysis stratified by study design in ITT analysis**

**
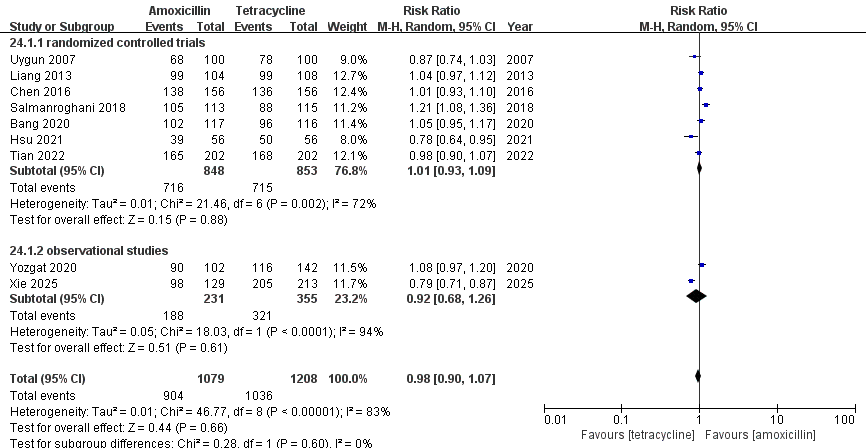
**

**Figure S2. Forest plot of subgroup analysis stratified by study design in PP analysis**

**
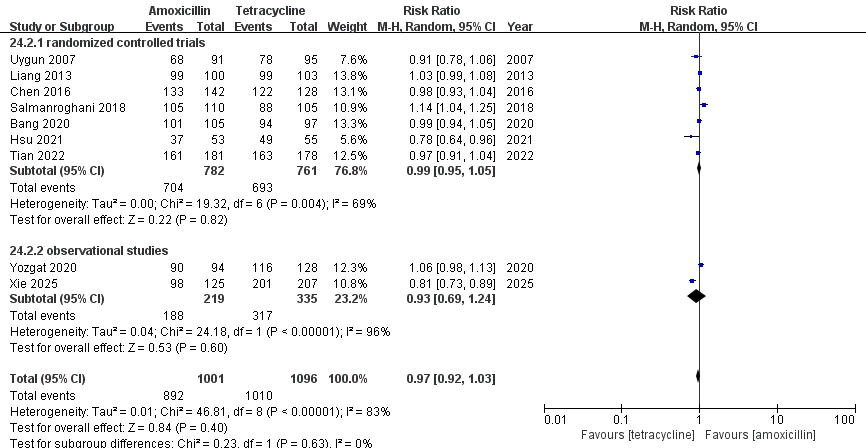
**

**Figure S3. Forest plot of subgroup analysis stratified by amoxicillin resistance**


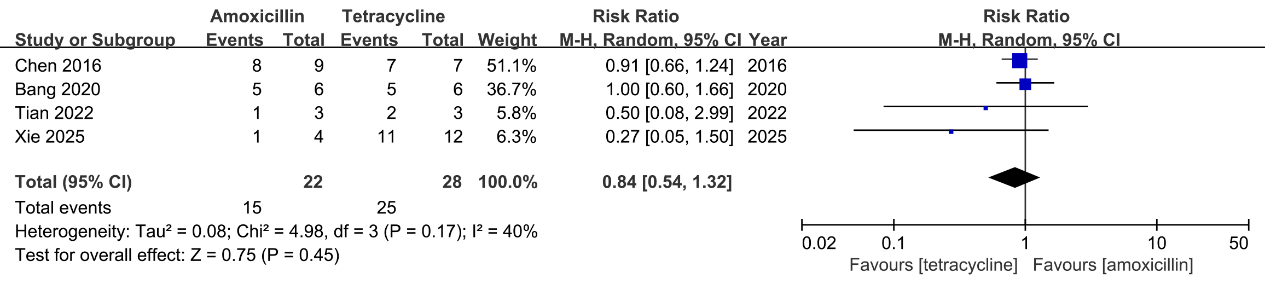


**Figure S4. Forest plot of subgroup analysis stratified by tetracycline resistance**

**
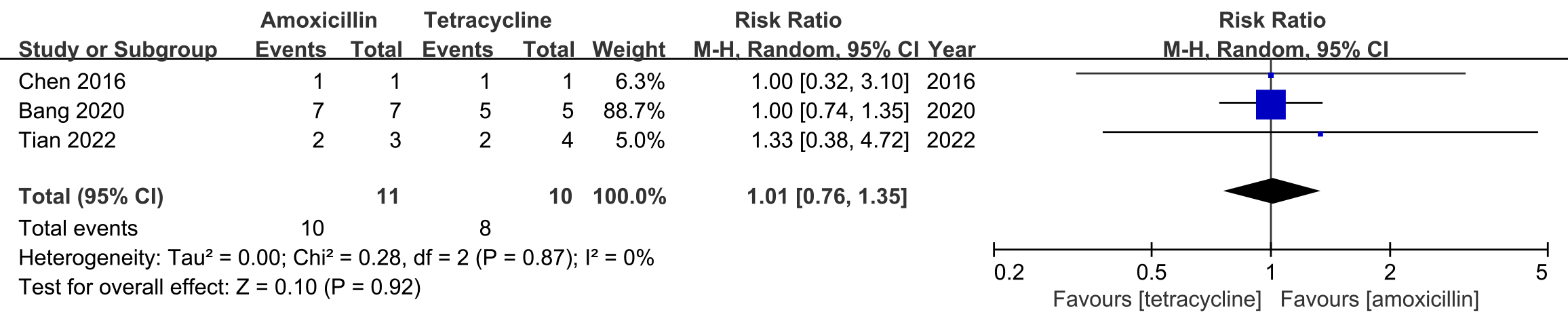
**

**Figure S5. Forest plot of subgroup analysis stratified by** **metronidazole resistance**


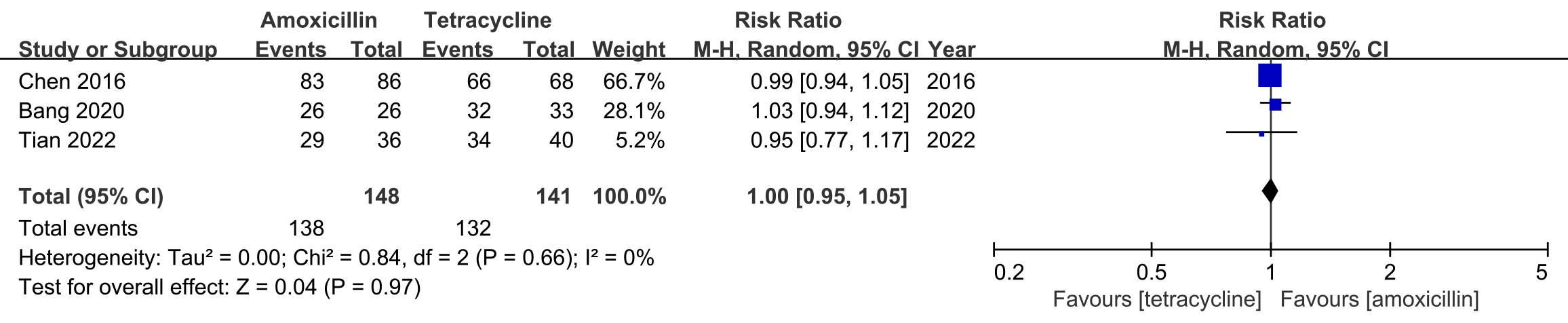


**Figure S6. Forest plot of subgroup analysis stratified by levofloxacin resistance**


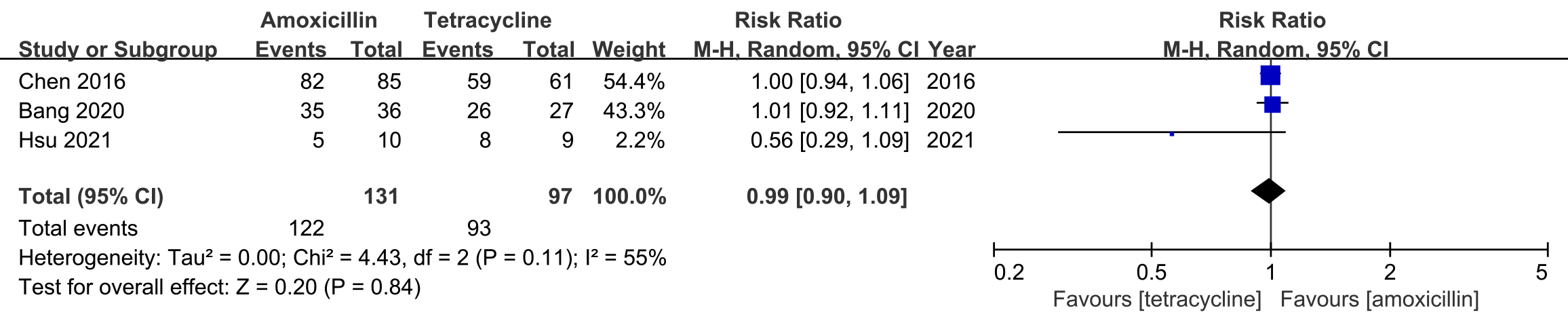


**Figure S7. Forest plot of subgroup analysis stratified by** **clarithromycin resistance**


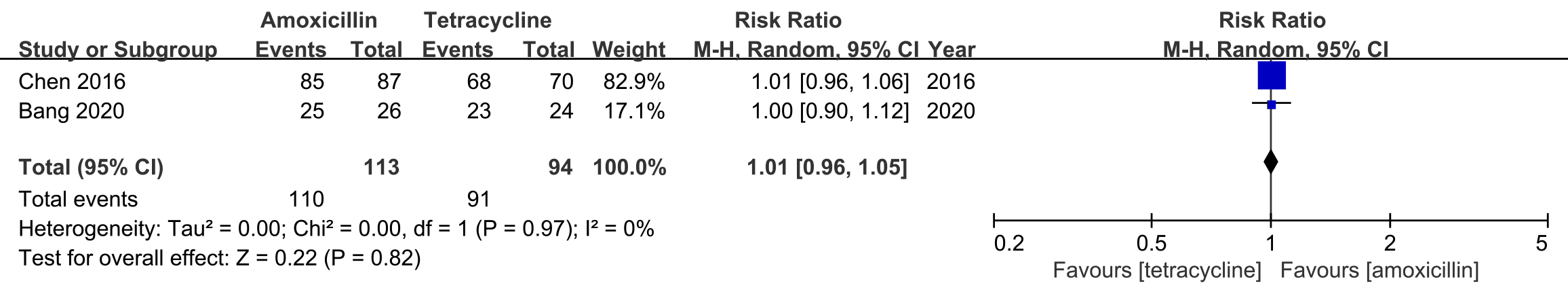


**Figure S8. Forest plot for the incidence of nausea/vomiting between amoxicillin-containing therapy and tetracycline-containing therapy**

**
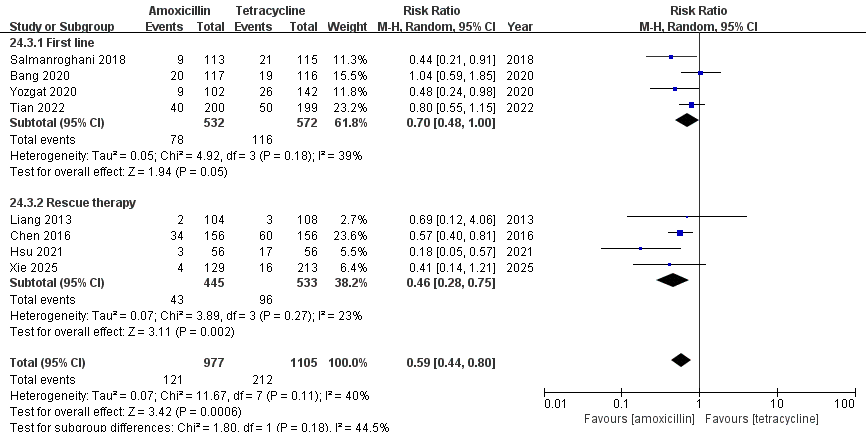
**

**Figure S9. Forest plot for the incidence of dizziness between amoxicillin-containing therapy and tetracycline-containing therapy**

**
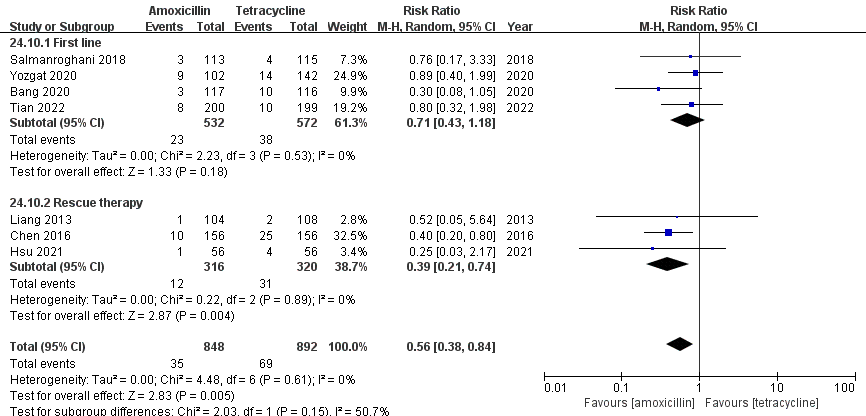
**

**Figure S10. Forest plot for the incidence of abdominal pain between amoxicillin-containing therapy and tetracycline-containing therapy**

**
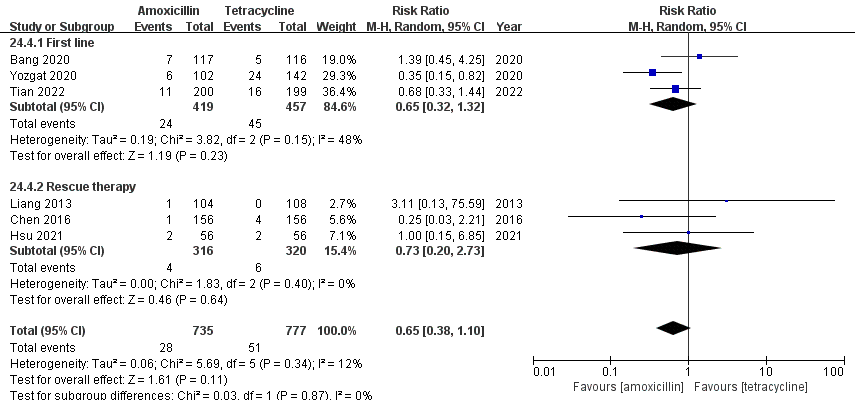
**

**Figure S11. Forest plot for the incidence of diarrhea between amoxicillin-containing therapy and tetracycline-containing therapy**

**
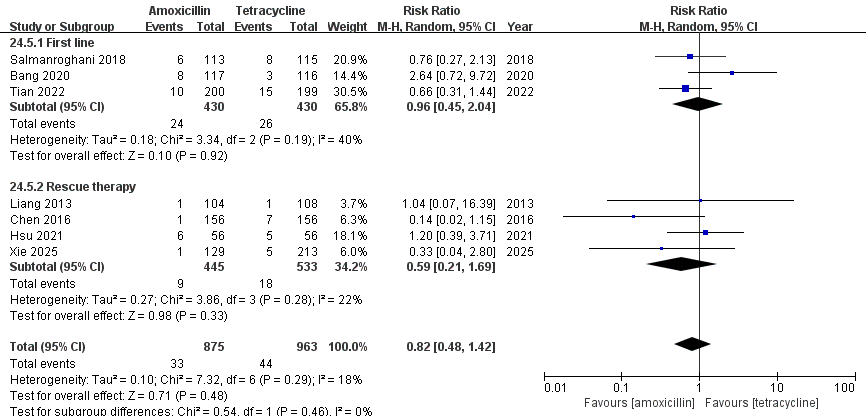
**

**Figure S12. Forest plot for the incidence of abnormal taste between amoxicillin-containing therapy and tetracycline-containing therapy**

**
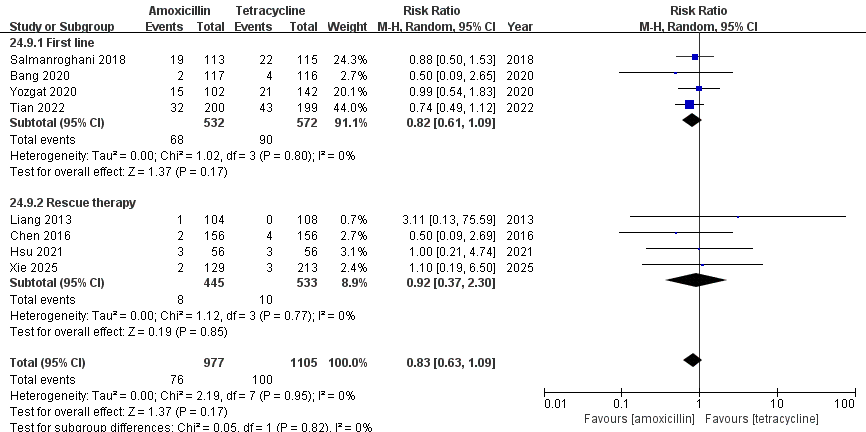
**

**Figure S13. Forest plot for the incidence of headache between amoxicillin-containing therapy and tetracycline-containing therapy**

**
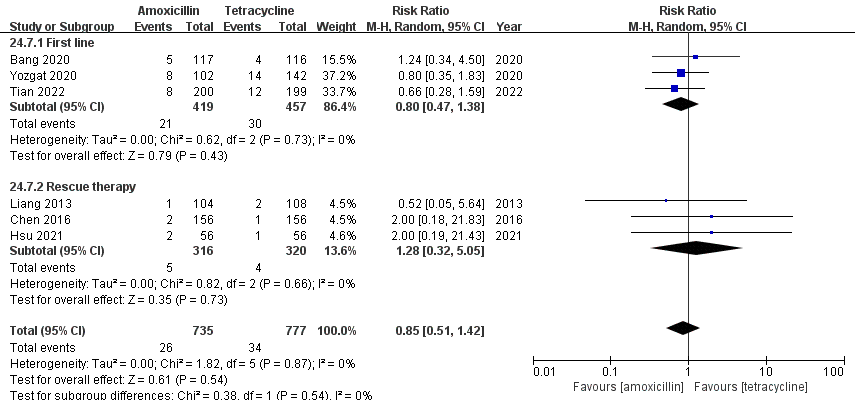
**

**Figure S14. Forest plot for the incidence of fatigue between amoxicillin-containing therapy and tetracycline-containing therapy**

**
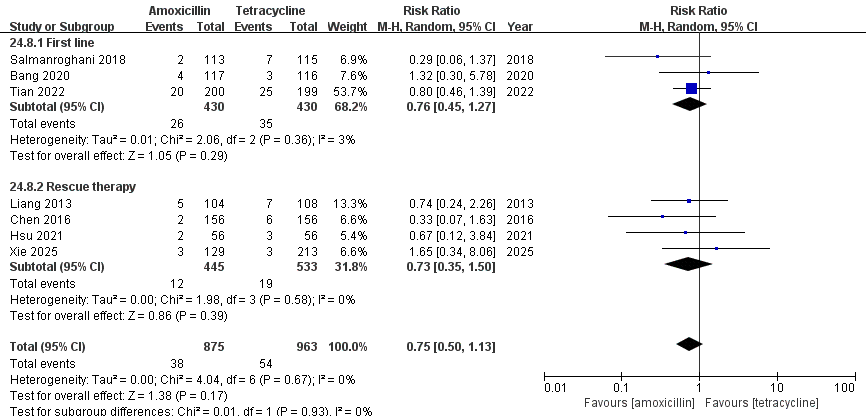
**

**Figure S15. Forest plot for the incidence of skin rash between amoxicillin-containing therapy and tetracycline-containing therapy**

**
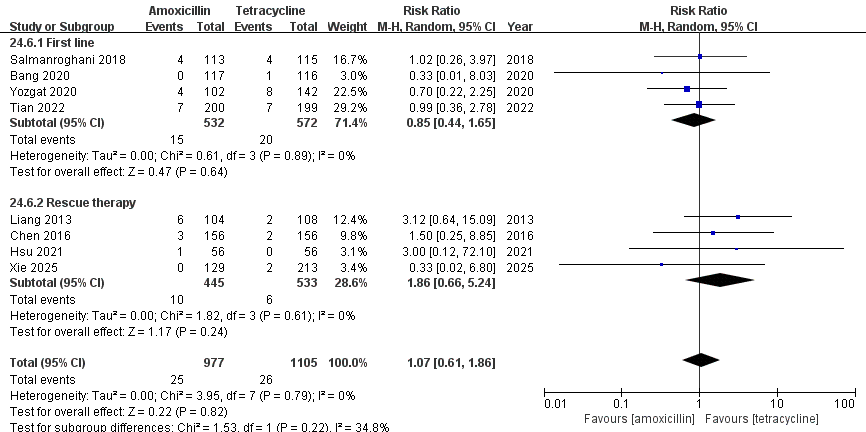
**
